# 

**Published:** 1881-01

**Authors:** 


### CONTENTS:

ART. I.—Proceedings of the Maryland and District of Columbia Dental Association. Annual Meeting.......................................385
Restoration of Contour as a Means of Separating Enamel Margins and Preventing Recurrence of Decay. By Marshall Webb, D. D. S..........392
ART. II.—Correspondence between Dr. T. B. Gunning and the Census Bureau......400
ART. III.—The Vulcanite Company vs. the Celluloid Co..........................410
ART. IV.—Irregularities of the Teeth and their Surgical Treatment. By Francis Fox.......................................419
Ohio State Dental Society..........................425
American Academy of Dental Science..........................426

### EDITORIAL, ETC.

The Goodyear Dental Vulcanite Company..........................427

Maryland Dentists and the Vulcanite Patent..........................427

Diamond Instruments..........................428

### BIBLIOGRAPHICAL.

Walsh's Physician's Call Book..........................429

### MONTHLY SUMMARY.

Alloys and Solders..........................429
The Tasting Power of the Tongue..........................430
Cervico-Bronchial Neuralgia Cured by Extraction of Wisdom Tooth..........................431
Anæsthesia by Rapid Respiration..........................431
Fetid Sweating of the Feet..........................432
Keeping Rubber Plates Clean..........................432
The Dangers of Chloroform..........................432

